# Sex and Sleep: Perceptions of Sex as a Sleep Promoting Behavior in the General Adult Population

**DOI:** 10.3389/fpubh.2019.00033

**Published:** 2019-03-04

**Authors:** Michele Lastella, Catherine O'Mullan, Jessica L. Paterson, Amy C. Reynolds

**Affiliations:** ^1^Appleton Institute for Behavioural Science, Central Queensland University, Rockhampton, QLD, Australia; ^2^School of Health, Medical and Applied Sciences, Central Queensland University, Rockhampton, QLD, Australia

**Keywords:** bedtime, orgasm, masturbation, gender, behavior, sex

## Abstract

**Objective:** The main aim of this study was to explore the perceived relationship between sexual activities, sleep quality, and sleep latency in the general adult population and identify whether any gender differences exist.

**Participants/methods:** We used a cross-sectional survey to examine the perceived relationship between sexual activity and subsequent sleep in the general adult population. Seven-hundred and seventy-eight participants (442 females, 336 males; mean age 34.5 ± 11.4 years) volunteered to complete an online anonymous survey at their convenience.

**Statistical Analyses:** Chi square analyses were conducted to examine if there were any gender differences between sexual activities [i.e., masturbation (self-stimulation), sex with a partner without orgasm, and sex with a partner with orgasm] and self-reported sleep.

**Results:** There were no gender differences in sleep (quality and onset) between males and females when reporting sex with a partner [$\chi_{\left(2\right)}^{2}$ = 2.20, *p* = 0.332; $\chi_{\left(2\right)}^{2}=$5.73, *p* = 0.057] or masturbation (self-stimulation) [$\chi_{\left(2\right)}^{2}$ = 1.34, *p* = 0.513; $\chi_{\left(2\right)}^{2}$ = 0.89, *p* = 0.640] involved an orgasm.

**Conclusions:** Orgasms with a partner were associated with the perception of favorable sleep outcomes, however, orgasms achieved through masturbation (self-stimulation) were associated with the perception of better sleep quality and latency. These findings indicate that the public perceive sexual activity with orgasm precedes improved sleep outcomes. Promoting safe sexual activity before bed may offer a novel behavioral strategy for promoting sleep.

## Introduction

Sleep problems are common and costly worldwide (1, 2). In particular, population estimates predominantly from Western countries have shown downward trends in average sleep duration and higher prevalence of insomnia and other sleep difficulties (3–5). While numerous studies have considered behavioral and lifestyle strategies to improve sleep onset, little is known about the relationship between a common bedtime activity: sexual behavior and sleep. Given that both sex and sleep are essential for the maintenance of physiological and psychological well-being, surprisingly few studies have explored the possibility that sexual activities may be associated with better quality sleep (6–9). The combined release of oxytocin, prolactin, and the inhibition of cortisol following orgasm may prompt a sleep facilitatory effect (10). Oxytocin is elevated as a result of sexual intercourse (11) and has been associated with a better quality of life, a reduction in stress (cortisol) and improved sleep quality in both males and females (12, 13). Prolactin, which is associated with both quality of orgasm and sexual satisfaction, has also been shown to increase following orgasm, and even more so, when orgasm occurs during sexual intercourse (10, 14). Together, these findings suggest that sexual activity may be part of the underlying neuro-hormonal mechanism facilitating sleep after sexual intercourse.

Brissette et al. (7) is the only investigation to examine sleep in humans following sexual activities with and without orgasm using the gold standard in sleep monitoring, polysomnography. This particular investigation examined the effects of solo masturbation on sleep latency and sleep architecture in five men and five women following masturbation with and without orgasm. While their findings revealed no differences for sleep latency or duration between genders across three conditions; no masturbation (involved light reading in bed), masturbation without orgasm, and masturbation with orgasm, some limitations within the experimental design may have affected their results. First, the presence of a researcher having to remove the anal probe following orgasm may have delayed participants' sleep latency. Second, the control condition required participants to read for 15 min before attempting sleep; this may have sleep inducing effects obscuring the results of this study. Finally, the findings of this study are limited to solo masturbation meaning less is known about the potential sleep benefits of sexual activity with a partner. Consequently, the relationship between sexual activities, sleep quality, and sleep latency is largely unknown in human subjects.

This warrants greater attention, as sexual activities followed by orgasm may have a facilitatory effect on human sleep (15–17) and thereby offer non-pharmacological alternative toward improving sleep. While, Kinsey et al. (9), frequently reported relaxation, sleepiness, and sleep onset as an aftereffect of orgasm in both male and females, Halpern and Sherman (16) only reported this facilitatory effect in males. Despite the early findings of Kinsey, little is known today about public perceptions of the relationship between sex, sleep quality, and sleep latency. Therefore, the main aim of this study was to explore the perceived relationship between sexual activities, sleep quality, and sleep latency in the general adult population and identify whether any gender differences exist.

## Participants and Methods

### Participants

Seven-hundred and seventy-eight participants aged 18 years and over (442 females, 336 males; mean age 34.5 ± 11.4 years) volunteered to complete an online anonymous survey at their convenience between October 2016 to June 2017. Informed consent was obtained from all individual participants included in the study. Participants were recruited through social media platforms (i.e., Twitter, Facebook) and professional networks. A link to the online survey was posted and participants were encouraged to repost as a form of snowballing sampling. Ethical approval was obtained through the University Human Research Ethics Committee (H16/09-260). All participants gave written informed consent in accordance with the Declaration of Helsinki.

### Instrument

The survey instrument included pre-validated items derived from the Australian Study of Health and Relationships (18) and the Pittsburgh Sleep Quality Index (19). The survey contained 49 questions relating to sleep/wake, sexual behaviors and demographic information such as age, gender, sexual orientation and relationship status.

### Demographic Questions

The first section was used to obtain demographic information about the participant (Table 1). Typical questions included: “*What is your age in years?,” “Gender,” “What is your relationship status,” and “What is your sexual identity (how one identifies one's sexual orientation).”*

**Table 1:** Characteristics of the sample (*n*, %) of participants (female and male).

**Characteristic**	**Total**		**Female**		**Male**	
**Age**	***n***	**%**	***n***	**%**	***n***	**%**
18–24	154	20.0	101	23.0	53	16.1
25–34	312	40.5	191	43.4	121	36.7
35–44	149	19.4	83	18.9	66	20.0
45–54	108	14.0	52	11.8	56	17.0
55+	47	6.1	13	3.0	34	10.3
**Sleep**	***n***	**M ±*****SD***	***n***	**M ±*****SD***	***n***	**M ±*****SD***
Bed time (hh:mm)			442	22:39 ± 2. 2.1	336	22:33 ± 2.4
Sleep latency (min)			442	27.5 ± 27.4	336	24.7 ± 37.5
Wake-up time (hh:mm)			442	07:08 ± 2.1	336	06:50 ± 1.9
Total sleep time (hours)	703	6.7 ± 1.6	400	6.8+1.3	303	6.7+1.2
**Sleep quality (PSQI)**	***n***	**%**	***n***	**%**	***n***	**%**
Very good	83	11.6	38	9.3	45	14.5
Fairly good	438	61.0	244	59.8	194	62.6
Fairly bad	169	23.5	107	26.2	62	20.0
Very bad	28	3.9	19	4.7	9	2.9
**Sexual orientation**						
Heterosexual	629	92.1	347	89.7	282	95.3
Homosexual	12	1.8	5	1.3	7	2.4
Bisexual	41	6.0	35	9.0	6	2.0
Undecided	1	0.1	0	0.0	1	0.3
**Relationship status**						
Single	104	13.4	61	13.8	43	12.8
De facto	77	9.9	54	12.2	23	6.8
Married	310	39.8	148	33.5	162	48.2
Partner	248	31.8	154	34.8	94	28.0
Separated/divorced	20	2.6	14	3.2	6	1.8
Other	19	2.4	11	2.5	8	2.4
**Sexual relationship type**						
Regular partner (live together)	474	69.5	259	67.1	215	72.6
Regular partner (live separately)	102	15.0	68	17.6	34	11.5
Occasional partner	38	5.6	24	6.2	14	4.7
Casual (one night stand)	21	3.1	7	1.8	14	4.7
No partner	47	6.9	28	7.3	19	6.4

### Sleep Variables

Research suggests that individuals have difficulty in estimating their “habitual” or average sleep duration, and consistently underestimate their total sleep duration (20). Sleep history, in the form sleep onset and offset has been shown to be a more precise measure of sleep duration (21). In line with the widely established Pittsburgh Sleep Quality Index participants were asked to assess sleep/wake behaviors “*over the past month”* to avoid the limitations of post sleep questions that may only assess the previous night's sleep see Buysse et al. (19). Typical questions included but were not limited to: “*During the past month, what time have you usually gone to bed at night (am/pm)?,” “During the past month, how long (in minutes) has it usually taken you to fall asleep each night?,” “During the past month, how many hours of actual sleep did you get at night (this may be different to the number of hours you spent in bed)?,” “During the past month, how would you rate your sleep quality overall?”*

### Sexual Behavior Variables

The third section consisted of a series of questions adapted from Richters et al. (18) related to participants' sexual behaviors, in terms of type of sexual activity and frequency. Sex was defined as sexual intercourse (vaginal or anal), oral sex, or manual stimulation of the genitals by a partner. The term sex did not include masturbation (self-stimulation). In this paper, we adopt this definition of sex. Likewise, masturbation was defined as self-stimulation of the genitals while alone. The final section combined questions related to both sexual and subsequent sleep behaviors. Typical questions and responses included but were not limited to:

“*On times when you had sex before attempting to go to sleep, do you feel this affected your sleep? Response: Yes, my sleep improved compared to average; No, my sleep stayed about the same as average; Yes, my sleep worsened compared to average.”*

“*On times when you have reached an orgasm with a partner, do you feel this affected your sleep? Response: Yes, my sleep improved compared to average; No, my sleep stayed about the same as average; Yes, my sleep worsened compared to average.”*

“*On times when you have masturbated before attempting to go to sleep, do you feel this affected your sleep? Response: Yes, my sleep improved compared to average; No, my sleep stayed about the same as average; Yes, my sleep worsened compared to average.”*

“*On times when you have reached an orgasm by yourself, do you feel this affected your sleep?*,

Response: Yes, my sleep improved compared to average; No, my sleep stayed about the same as average; Yes, my sleep worsened compared to average.”

### Honesty and Embarrassment

As content in the questionnaire was of a highly personal nature and there is the possibility of influence over responses, participants were asked specific questions at the end to determine the honesty of responses. Questions were adapted from the Australian Study of Health and Relationships (21). How embarrassing did you find the survey? With response items *Extremely, Very, Quite, Slightly, Not at all*. In percentage terms, how honest were you in your answers to the Questionnaire?

### Analysis

Descriptive statistics were conducted to explore the sleep/wake behaviors and frequency of sexual activity for all participants. Chi square analyses were conducted to examine if there were any gender differences between sexual activities (i.e., masturbation, sex with a partner without orgasm, and sex with a partner with orgasm) and self-reported sleep. All statistical analyses were computed using SPSS Statistics (v25, IBM, USA).

## Results

A total of 778 participants completed the survey and provided information on their gender (56.8% female). A total of 683 (87.8%) of these participants provided information about sexual orientation. Of those who responded, responses reflected a high proportion of participants who identified as heterosexual orientation (*n* = 629, 92.1%); consequently, perceptions of the relationship between sexual activity before bed and sleep factors are reported by gender differences only. Characteristics of participants are provided in Table 1.

### Perceptions of the Relationship Between Sexual Activity Before Bed and Sleep Factors

Despite the sensitive nature of the topic, between 505 and 582 participants (64.9–74.8% response rate; see Table 2) provided complete responses on their perception of sexual activity before bed and sleep factors. While some participants may have elected not to answer due to sensitive content, or discomfort, the response rate is unlikely to be solely a consequence of this. Of the 613 (78.8%) of participants who responded to embarrassment and honesty indicators at the conclusion of the survey, the majority of the sample found the questions regarding sex and sleep slightly (*n* = 134; 21.9%) or not at all (*n* = 381; 62.2%) embarrasing. A small proportion of the sample indicated they felt extremely (*n* = 13; 2.1%), very (*n* = 33; 5.4%), or quite (*n* = 52; 8.5%) embarrassed. These feelings did not appear to influence honesty of responses, with a mean(±SD) honesty score of 99.5 ± 37.2 out of a possible 100.

**Table 2:** Participant perceptions on the relationship between sexual activity before sleep and sleep factors.

		**Total**		**Female**		**Male**		**p**
		**n**	**%**	**n**	**%**	**n**	**%**	
**SEX WITH A PARTNER BEFORE SLEEP**								
**Sleep quality**	Improves	367	63.1	192	59.1	175	68.1	0.026
	Stays the same	190	32.6	114	35.1	76	29.6	
	Worsens	25	4.3	19	5.8	6	2.3	
		582	100.0%	325	100.0%	257	100%	
**Sleep onset**	Improves	341	59.0	172	53.3	169	66.3	0.001
	Stays the same	189	32.7	114	35.3	75	29.4	
	Worsens	48	8.3	37	11.4	11	4.3	
		578	100%	323	100.0%	255	100.0%	
**ORGASM WITH A PARTNER BEFORE SLEEP**								
**Sleep quality**	Improves	409	70.8	221	68.4	188	73.7	0.332
	Stays the same	154	26.6	92	28.5	62	24.3	
	Worsens	15	2.6	10	3.1	5	2.0	
		578	100.0%	323	100.0%	255	100.0%	
**Sleep onset**	Improves	357	62.5	186	58.3	171	67.6	0.057
	Stays the same	181	31.6	110	34.5	7	28.1	
	Worsens	34	5.9	23	7.2	11	4.3	
		572	100.0%	319	100.0%	189	100.0%	
**MASTURBATION BEFORE SLEEP**								
**Sleep quality**	Improves	246	48.2	138	49.8	108	46.4	0.734
	Stays the same	251	49.2	132	47.7	119	51.1	
	Worsens	13	2.6	7	2.5	6	2.5	
		510	100.0%	277	100.0%	233	100.0%	
**Sleep onset**	Improves	226	44.7	125	46.0	101	43.3	0.839
	Stays the same	254	50.3	134	49.2	120	51.5	
	Worsens	25	5.0	13	4.8	12	5.2	
		505	100.0%	272	100.0%	233	100%	
**ORGASM WITH MASTURBATION BEFORE SLEEP**								
**Sleep quality**	Improves	284	54.1	158	55.8	126	52.0	0.513
	Stays the same	231	44.0	121	42.8	110	45.5	
	Worsens	10	1.9	4	1.4	6	2.5	
		525	100.0%	283	100.0%	242	100.0%	
**Sleep onset**	Improves	245	47.4	132	47.0	113	47.9	0.640
	Stays the same	248	48.0	138	49.1	110	46.6	
	Worsens	24	4.6	11	3.9	13	5.5	
		517	100.0%	281	100.0%	236	100.0%	

### Sex With a Partner Before Sleep: Differences With and Without Orgasm

Perceived relationship between sex with and without orgasm, and masturbation with and without orgasm, with sleep factors are reported in Table 2. Overall, a large percentage of respondents perceived there were improvements in their sleep quality (*n* = 367; 63.1%) and sleep onset (*n* = 341; 59.0%) when they had sex with a partner before sleep. There were significant gender differences in this perception ($\chi_{\left(2\right)}^{2}$ = 7.30, *p* = 0.026, see Table 2), with a higher percentage of male respondents (68.1%) than female respondents (59.1%) indicating that they perceived their sleep quality improved when they had sex with a partner before sleep. A similar gender difference was observed in perceived sleep onset ($\chi_{\left(2\right)}^{2}$ = 14.36, *p* = 0.001, with a greater percentage of male participants (66.3%) indicating they perceived sex with a partner before sleep improved their sleep onset compared to 53.3% of female respondents. A higher percentage of female participants (11.4%) perceive that sex with a partner before sleep worsens their sleep onset compared with male participants (4.3%).

A higher percentage of participants overall felt that their sleep quality (70.8%) and sleep onset (62.5%) improved after achieving orgasm with a partner before bed. No significant gender differences were apparent in responses for either sleep quality ($\chi_{\left(2\right)}^{2}$ = 2.20, *p* = 0.332) or sleep onset ($\chi_{\left(2\right)}^{2}$ = 5.73, *p* = 0.057; see Table 2).

### Masturbation Before Sleep: Differences With and Without Orgasm

Descriptive statistics indicated that the percentage of participants who perceived masturbation improved sleep quality (48.2%) and sleep onset (44.7%) was lower than the perceived effect of sex with a partner. Percentages increased slightly when participants were asked about orgasm with masturbation, with 54.1% of participants reporting improved sleep quality, and 47.4% reporting improved sleep onset. No gender differences were found in perceptions of the impact of masturbation on sleep quality ($\chi_{\left(2\right)}^{2}$ = 0.62, *p* = 0.734) or sleep onset ($\chi_{\left(2\right)}^{2}$ = 0.35, *p* = 0.839). Similarly, no gender differences were found in perceived impact of masturbation with orgasm on sleep quality ($\chi_{\left(2\right)}^{2}$ = 1.34, *p* = 0.513) or sleep onset ($\chi_{\left(2\right)}^{2}$ = 0.89, *p* = 0.640).

## Discussion

This study is the first to explore the perceived relationship between sexual activities, sleep quality and sleep latency in the general adult population to specifically identify whether any gender differences exist. A difference between males' and females' perceptions of sleep quality, particularly following sex with a partner, was apparent. Significant gender differences existed in perception of sex with a partner and impact on subsequent sleep quality and sleep latency. Specifically, a higher proportion of males reported perceived improvement in sleep quality and sleep following sex with a partner. The reason for the difference between males and females was not explored in this study, but it may be explained by the gender gap in orgasm frequency; that is, men, when compared with women, are more likely to orgasm during sex with a partner (22). The wording of the survey question regarding sex with a partner, however, may not have adequately captured the nuances of this situation, and should be considered in future studies.

Most notably, there were no differences in perceived sleep quality or latency between males and females when sex with a partner involved an orgasm, with the majority of both men and women indicating they felt sleep quality and latency improved with orgasm. These findings seem to be consistent with the hypothesis put forward by Brody and Krüger (10) that higher levels of oxytocin and prolactin following orgasm may prompt a sleep facilitatory effect. Furthermore, our findings appear to corroborate the ideas of Leeners et al. (14) linking higher levels of sexual satisfaction and quality of orgasm with increased levels of prolactin in women. Only 3–6% of all participants indicated that they felt they slept worse following sex that involved a partner and an orgasm. These findings suggest that sex with a partner involving an orgasm may serve as a means to promote and improve sleep for both genders.

Interestingly, no gender differences were found in perceptions of the impact of masturbation on sleep quality or sleep onset with or without orgasm. This finding broadly supports the work of Brissette et al. (7) who also found no gender differences in sleep outcomes resulting from solo masturbation. Our findings did, however, indicate that over 50% of participants had improved sleep quality through masturbation resulting in an orgasm. This further supports the idea of orgasm, as opposed to sexual activity, facilitating sleep in both men and women.

## Strengths and Limitations

While the present study was unable to capture participants' physiological responses following sexual behavior (either through sex or masturbation), it is the first cross-sectional survey to explore the relationship between sexual behavior and perceptions of subsequent sleep, and to identify whether any gender differences exist. The study was strengthened by a large sample of individuals with a relatively even balance between gender (57% female; 43% male). The inclusion of sex with a partner and solitary sexual activity (masturbation) allowed a more accurate understanding of the nuances around sexual activity. Despite the sensitive nature of the topic, the majority of the sample reported answering the questions honestly. There are some limitations that need to be acknowledged. The completion rate among respondents was 66% indicating that a non-response bias may be an issue. Consistent with previous sexuality research (18), the present study relied on self-reported perceptions of sexual behaviors and sleep which may be affected by desirability and/or recall bias. In addition, the use of a cross-sectional survey restricted our ability to imply causations for the associations identified.

## Future Directions and Conclusions

The results of this study indicate that when sexual activities culminate in an orgasm, there are no gender differences in terms of perceptions of the impact on sleep. Whilst orgasms with a partner appear to have the most benefit in terms of sleep outcomes, orgasms achieved through self- stimulation can also aid sleep quality and latency. Engaging in safe and satisfying sexual activity (either alone or with a partner) together with other sleep hygiene strategies before attempting sleep, may offer the general adult population a healthy behavioral approach toward improving their subsequent sleep. Moving forward, it is important to recognize that sexual activity is a taboo topic and not widely discussed within many social arenas (23). If we are to adequately understand the causal relationship between sexual activity and sleep, future investigations need to examine participants' physiological responses following sexual behavior. In addition, efforts to reduce the stigma associated with this topic are needed.

## Data Availability

The datasets generated for this study are available on request to the corresponding author.

## Author Contributions

ML led the overall study, contributed to the data collection and interpretation, and wrote the manuscript. AR contributed to the data collection, data analysis, and manuscript edits. CO contributed to the data interpretation and manuscript edits. JP contributed to study design and manuscript edits. All authors read, contributed to the research design, and approved the final manuscript.

### Conflict of Interest Statement

The authors declare that the research was conducted in the absence of any commercial or financial relationships that could be construed as a potential conflict of interest.
